# The complete mitochondrial genome of the *Cyathochromis obliquidens*

**DOI:** 10.1080/23802359.2017.1334521

**Published:** 2017-06-13

**Authors:** Xiangru Wen, Cailin Wang, Man Li, Haiyan Wang, Dashi Qi

**Affiliations:** School of Basic Education Sciences, Xuzhou Medical University, Xuzhou, Jiangsu, China

**Keywords:** Mitochondrial genome, *Cyathochromis obliquidens*, phylogenic relationship

## Abstract

The *Cyathochromis obliquidens*, the only member of *Cyathochromis* genus, is widely spread in Africa. In this study, we firstly reported the complete mitochondrial genome of *C. obliquidens*. The whole mitochondrial genome is 16,581 bp in length, including 2 ribosomal RNA genes, 22 transfer RNA genes and 13 protein-coding genes. Its GC content is 45.94%, similar to the other species from the same family, like *Alticorpus geoffreyi* (45.82%). We also analyzed the complete mitochondrial genome of *C. obliquidens* and its phylogenic relationship with other 14 related species, which would help our better understanding of the evolution of Cichlidae mitochondrial genome.

As a member of Cichlidae family, the *Cyathochromis obliquidens* is widely spread in Africa (Meyer et al. 2015). Here, we firstly reported the complete mitochondrial genome of *C. obliquidens*, which would facilitate our understanding of the mitochondrial genome and the phylogenic relationship of the Cichlidae family.

In this study, we assembled the complete mitochondrial genome of *C. obliquidens* (Genbank accession: MF033354) based on the raw data of the whole genome of a *C. obliquidens* (SRA: ERP002088). The *C. obliquidens* sample (Sample ID: SAMEA2661259) was collected from a trawler catch in the Southeast Arm of Lake Malawi and sequenced by the Wellcome Trust Sanger Institute (SC) in their Cichlid diversity sequencing WTMGM student project.

By using SOAPaligner/soap2 (V2.21) (Li et al. 2009), we mapped all the raw reads to the reference genome, complete mitochondrial genome of *Alticorpus geoffreyi* (Genbank accession: NC_028033) (Qi et al. 2016). We assembled these reads which could map to the reference genome by SPAdes3 (V3.1.0) (Bankevich et al. 2012), and got the complete circular mitochondrial genome of *C. obliquidens*. Also, we used DOGMA(Wyman et al. 2004) and tRNAscan-SE 2.0 (Lowe & Eddy 1997) to annotate the complete mitochondrial genome.

The mitochondrial genome of *C. obliquidens* is 16,581 bp in length, contains 37 genes, including 2 ribosomal RNA genes (rRNA), 22 transfer RNA genes (tRNA) and 13 protein-coding genes (PCGs). The lengths of 22 tRNA genes range from 67 bp (tRNA^Cys^ and tRNA^Ser^) to 74 bp (tRNA^Leu^ and tRNA^Lys^), whereas 16S rRNA is 1676 bp and 12S rRNA is 941 bp. Twenty-eight genes contain 2 rRNA, 14 tRNA and 12 PCGs are H-strand, while the remaining 9 genes including 1 PCGs (ND6) and 8 tRNA are L-strand. All PCGs in *C. obliquidens* are started with ATG and stopped with TAN or AGA, except for COX1 started with GTG. The GC content of this mitochondrial genome is 45.94% (27.43% A, 26.63%T, 30.08% C, 15.86% G), similar to *A. geoffreyi* (45.82%) from the same family, Cichlidae.

Furthermore, we used MEGA7 (V7.0.25) (Kumar et al. 2016) to construct the phylogenetic tree on the complete mitochondrial genomes of *C. obliquidens*, 2 species with the same phylum, 2 species from the same class and other 10 closely related species by Maximum likelihood method (Figure 1). Those results would facilitate our understanding of the mitochondrial evolution of Cichlidae family.

**Figure 1.**
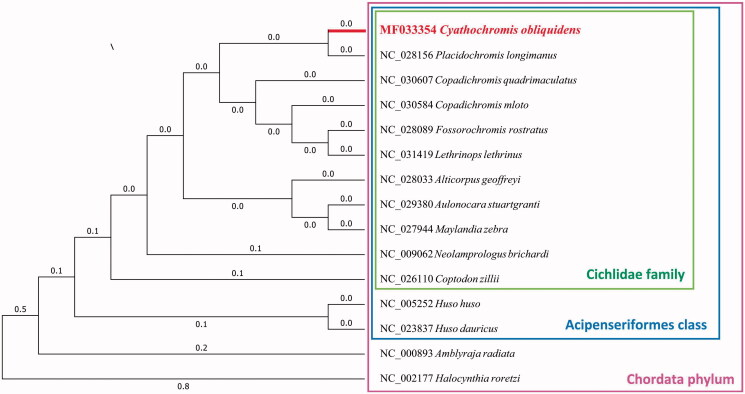
Maximum likelihood tree of complete mitochondrial genome of *C. obliquidens* and 14 other closely species, which have the Genbank accession numbers of their complete mitochondrial genome sequences in front.
